# Homozygosity Mapping and Targeted Sanger Sequencing Reveal Genetic Defects Underlying Inherited Retinal Disease in Families from Pakistan

**DOI:** 10.1371/journal.pone.0119806

**Published:** 2015-03-16

**Authors:** Maleeha Maria, Muhammad Ajmal, Maleeha Azam, Nadia Khalida Waheed, Sorath Noorani Siddiqui, Bilal Mustafa, Humaira Ayub, Liaqat Ali, Shakeel Ahmad, Shazia Micheal, Alamdar Hussain, Syed Tahir Abbas Shah, Syeda Hafiza Benish Ali, Waqas Ahmed, Yar Muhammad Khan, Anneke I. den Hollander, Lonneke Haer-Wigman, Rob W. J. Collin, Muhammad Imran Khan, Raheel Qamar, Frans P. M. Cremers

**Affiliations:** 1 Department of Biosciences, Commission on Science and Technology for Sustainable Development in the South Institute of Information Technology, Islamabad, Pakistan; 2 Department of Human Genetics, Radboud University Medical Center, Nijmegen, the Netherlands; 3 Tufts University Medical School, Boston, Massachusetts, United States of America; 4 Al-Shifa Eye Trust Hospital, Rawalpindi, Pakistan; 5 Department of Ophthalmology, Radboud University Medical Center, Nijmegen, the Netherlands; 6 Institute of Pure and Applied Biology, Bahauddin Zakariya University, Multan, Pakistan; 7 University of Haripur, Haripur, Pakistan; 8 Department of Chemistry, University of Science and Technology, Bannu, Pakistan; 9 Radboud Institute for Molecular Life sciences, Radboud University Nijmegen, Nijmegen, the Netherlands; 10 Al-Nafees Medical College & Hospital, Isra University, Islamabad, Pakistan; National Eye Institute, UNITED STATES

## Abstract

**Background:**

Homozygosity mapping has facilitated the identification of the genetic causes underlying inherited diseases, particularly in consanguineous families with multiple affected individuals. This knowledge has also resulted in a mutation dataset that can be used in a cost and time effective manner to screen frequent population-specific genetic variations associated with diseases such as inherited retinal disease (IRD).

**Methods:**

We genetically screened 13 families from a cohort of 81 Pakistani IRD families diagnosed with Leber congenital amaurosis (LCA), retinitis pigmentosa (RP), congenital stationary night blindness (CSNB), or cone dystrophy (CD). We employed genome-wide single nucleotide polymorphism (SNP) array analysis to identify homozygous regions shared by affected individuals and performed Sanger sequencing of IRD-associated genes located in the sizeable homozygous regions. In addition, based on population specific mutation data we performed targeted Sanger sequencing (TSS) of frequent variants in *AIPL1*, *CEP290*, *CRB1*, *GUCY2D*, *LCA5*, *RPGRIP1* and *TULP1*, in probands from 28 LCA families.

**Results:**

Homozygosity mapping and Sanger sequencing of IRD-associated genes revealed the underlying mutations in 10 families. TSS revealed causative variants in three families. In these 13 families four novel mutations were identified in *CNGA1*, *CNGB1*, *GUCY2D*, and *RPGRIP1*.

**Conclusions:**

Homozygosity mapping and TSS revealed the underlying genetic cause in 13 IRD families, which is useful for genetic counseling as well as therapeutic interventions that are likely to become available in the near future.

## Introduction

Inherited retinal diseases (IRD) refer to a clinically and genetically heterogeneous group of genetic eye disorders in which the photoreceptors and retinal pigment epithelium can be affected. There is an overlap of clinical features between different IRDs, which includes syndromic or non-syndromic conditions. In cone dystrophy (CD), only central vision is impaired, whereas in cone-rod dystrophy (CRD) peripheral vision is also compromised. In retinitis pigmentosa (RP) initially peripheral vision is affected, which later progresses to central vision defects. In contrast, congenital stationary night blindness (CSNB) only involves night vision loss due to defective rod photoreceptors. The most severe form of IRD is Leber congenital amaurosis (LCA), in which patients suffer from complete blindness in the first year of life [1–3]. In addition to clinical diversity, the genetic heterogeneity in IRDs is reflected by 221 genes that have thus far been found to be mutated in IRD (https://sph.uth.edu/retnet/). Besides clear phenotypic differences, different defects in the same gene may also be responsible for different clinical phenotypes, for example different variations in *RPGRIP1* (MIM # 605446) are known to cause RP, LCA and CRD, *TULP1* (MIM # 602280) mutations have been shown to cause RP, LCA or CD [4], and *RPGR* (MIM # 312610) variants are known to cause RP or CD [5]. It has also been observed that the inherited forms of retinal diseases follow all Mendelian modes of inheritance [3].

The prevalence of retinal dystrophies has been estimated at 1 in 3,000 individuals worldwide, with RP being the most common type affecting 1 in 4,000 individuals [6–8]. In Pakistan the prevalence of IRDs is not well defined but a hospital based study estimated that 1 in 800 patients who attended the ophthalmic outpatient department, were affected with retinal diseases, with RP as the most common phenotype [9]. However, such inherited disorders have been observed more commonly in consanguineous families than in non-consanguineous families. Hamamy et al. [10] calculated the percentage of the mode of inheritance of genetically inherited diseases and suggested that consanguinity is strongly correlated with the prevalence of autosomal recessive diseases. In addition, similar observations have been made by Bittles [11] and Nirmalan et al. [12]. In the Pakistani population more than 60% of marriages are consanguineous, and among them more than 80% are first cousin marriages [11]. For consanguineous IRD families with multiple affected individuals, the causative genetic defects can be identified using genome wide single nucleotide polymorphism (SNP)-array analysis followed by homozygosity mapping [13–15]. In view of the high genetic heterogeneity, homozygosity mapping in most isolated cases cannot unambiguously point to a single IRD-associated gene. Khan et al. [16] comprehensively reviewed the genetic causes of IRDs in the Pakistani population, and proposed an initial mutation screening method of IRDs by analyzing frequently occurring mutations. Since 2008, we have collected 81 consanguineous IRD families in Pakistan, and reported on the underlying genetic causes in 25 of these families [14,16–26].

In the current study we report the results from an additional 13 of these previously identified families. We performed genome-wide SNP genotyping followed by homozygosity mapping and candidate gene sequencing. In addition, we analyzed several families using targeted Sanger sequencing (TSS) of frequently reported variations from Pakistani population in *AIPL1* (MIM # 604392), *CEP290* (MIM # 610142), *CRB1* (MIM # 604210), *GUCY2D* (MIM # 600179), *LCA5* (MIM # 611408), *RPGRIP1* and *TULP1*.

## Materials and Methods

### Subjects

Since 2008, we have recruited 81 IRD families from different regions of Pakistan, i.e. CD (2 families), CSNB (5 families), LCA (36 families), and RP (38 families) (S1 Table).

### Ethics statement

The current study adheres to the declaration of Helsinki, and was approved by the Department of Biosciences Ethics Review Board of COMSATS Institute of Information Technology, Al-Shifa Eye Trust Hospital, Rawalpindi and Shifa International hospital, Islamabad. The subjects and their families were informed about the purpose of the study and their oral as well as written consent was taken.

### Clinical evaluations

The subjects were clinically diagnosed as CD, CSNB, LCA and RP on the basis of detailed ophthalmic evaluations and fundus examination. The affected individuals complaining of reduced central vision with focusing error, photophobia and nystagmus were grouped as CD. The individuals experiencing non-progressive night blindness with normal day vision were categorized as CSNB. The subjects were categorized as LCA if they were congenitally blind, had nystagmus, and sluggish or non-reactive pupilary response. Finally, the cases reporting night vision loss with progressive mid-peripheral vision deterioration were grouped as RP (S1 Table).

### DNA isolation

Blood samples were drawn from all available affected and unaffected individuals of the family into ethylenediamine tetra-acetic acid (EDTA)-coated vacutainers. DNA was extracted in Tris-EDTA buffer using a standard organic extraction protocol for 53 families and stored at −20°C. For the remaining 28 families, a standard salting out protocol was employed [27].

### Genetic linkage analysis

**Fig 1:**
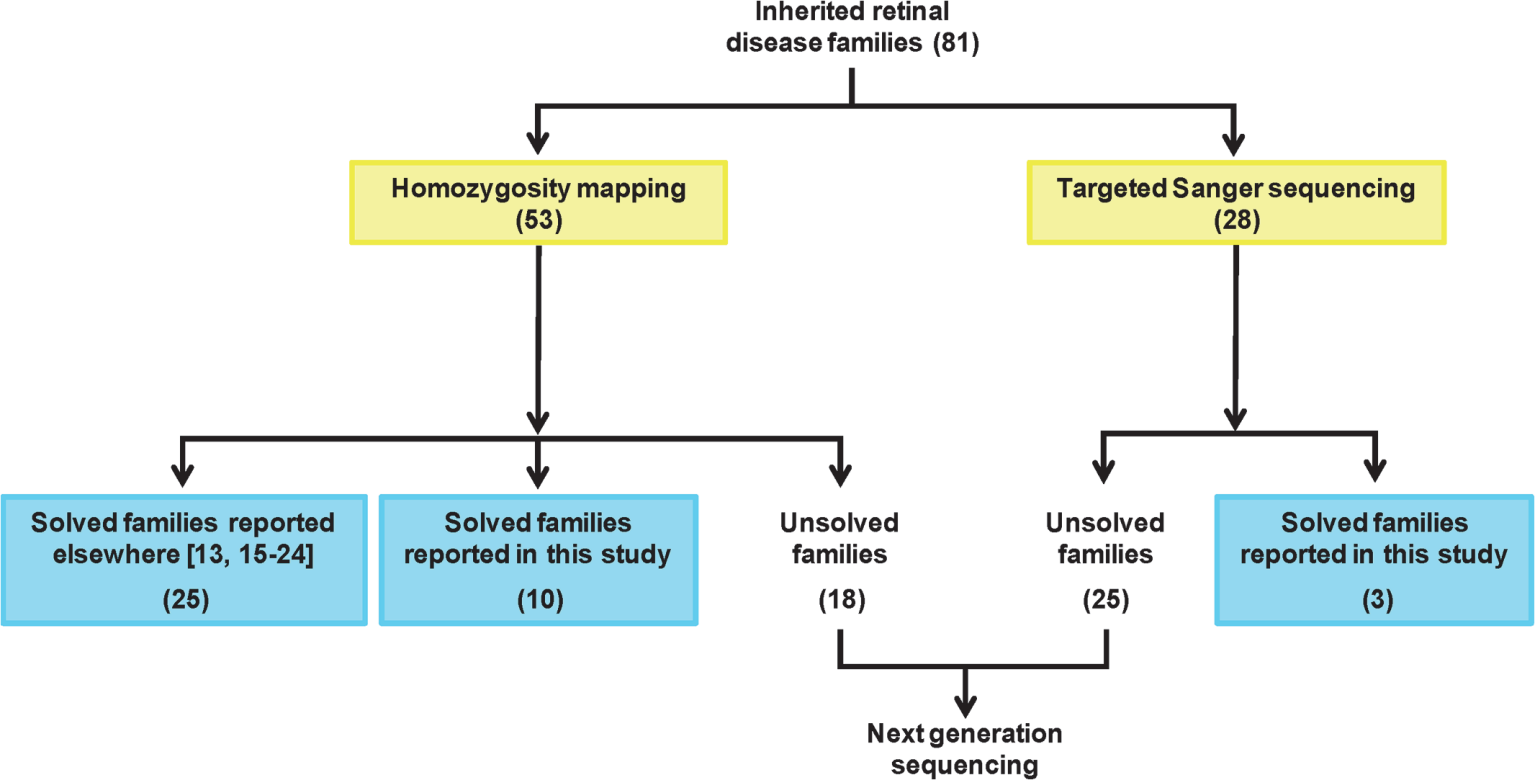
Workflow on Pakistani inherited retinal disease cohort. Numbers in parentheses indicate families.

Genetic linkage analysis was carried out for 53 of 81 families using microsatellite markers or whole genome SNP array platforms such as Illumina_10K, Affymetrix_6K, Human Omni express_700k and Cytoscan HD (Fig. 1, Table 1). The SNP array data were analyzed by homozygosity mapping using an online tool ‘Homozygosity Mapper’ (http://www.homozygositymapper.org/). Sanger sequencing was performed for IRD-associated genes. These genes were prioritized according to the size of the region in which they were located. First, any mutation hotspot in the gene, if known, was sequenced followed by sequencing of other exons along with flanking intronic sequences. Novel missense mutations were also screened in ethnicity-matched controls (n = 90).

**Table 1:** Results of targeted Sanger sequencing and genetic analyses of 13 IRD families.

Family ID	Disease	Genotyping method	Number of Hz regions	Size of Hz region, in Mb	Ranking of Hz region	Gene	DNA mutation	Predicted protein variant	First report of variant
**F01**	LCA	TSS	—	—	—	*AIPL1*	c.834G>A	p.(W278*)	[32]
**F02**	LCA	TSS	—	—	—	*AIPL1*	c.834G>A	p.(W278*)	[32]
**F03**	LCA	TSS	—	—	—	*GUCY2D*	c.2283del	p.(S762Afs*22)	This study
**F04**	LCA	Cytoscan HD, SS	5	14.0	1	*RPGRIP1*	c.3565C>T	p.(R1189*)	[31]
**F05**	LCA	Illumina_700K, SS	>10	4.5	19	*RPGRIP1*	c.930+1G>A	p.(?)	This study
**F06**	RP	Illumina_700K, SS	3	1.6	2	*RPE65*	c.131G>A	p.(R44Q)	[36]
**F07**	RP	Illumina_700K, SS	3	5.2	1	*RPE65*	c.361del	p.(S121Lfs*6)	[36]
**F08**	RP	Illumina_700K, SS	4	18.8	1	*CNGA1*	c.1298G>A	p.(G433D)	This study
**F09**	RP	Illumina_700K, SS	1	6.5	1	*CNGB1*	c.2493–2A>G	p.(?)	This study
**F10**	RP	Illumina_700K, SS	>10	13.4	2	*CRB1*	c.2234C>T	p.(T745M)	[38]
**F11**	RP	Illumina_700K, SS	>10	9.2	1	*TULP1*	c.1466A>G	p.(K489R)	[40]
**F12**	RP	Illumina_700K, SS	>10	6.5	1	*PDE6A*	c. 304C>A	p.(R102S)	[41]
**F13**	RP	Affymetrix 10K, SS	6	—	—	*RPGR*	c.2426_2427del	p.(E809Gfs*25)	[42]

Hz, Homozygous; Mb, Megabases; SS, Sanger sequencing entire gene; TSS, targeted Sanger sequencing; DNA, Deoxyribonucleic acid.

### Targeted Sanger sequencing (TSS)

In probands from 28 families diagnosed with LCA (from a total of 36), the targeted variant screening was done using Sanger sequencing (Table 2). The variants were chosen based on their frequency in the Pakistani population [16]. In addition, we also screened our LCA panel with other frequent variants that are associated with LCA in the Caucasian population including the intronic *CEP290* variant c.2991+1655A>G [28] and the *GUCY2D* exon 12 variant c.2302C>T [29,30]. The *RPGRIP1* variant (c.3565C>T) described by Abu-Safieh et al. [31] was found to be segregating in one of our LCA families (F04) and therefore this variant was also analyzed in our LCA cohort [16].

**Table 2:** Frequent variants pre-screened in 28 LCA families.

Gene	DNA variant	Protein variant	Reference
***AIPL1***	c.834G>A	p.(W278*)	[16,32,62,63]
***CRB1***	c.2234C>T	p.(T745M)	[38]
***CRB1***	c.2536G>A	p.(G846R)	[64]
***CRB1***	c.2966T>C^#^	p.(I989T)	[64]
***CEP290***	c.2991+1655A>G	p.(C998*)/WT^$^	[28]
***GUCY2D***	c.2302C>T	p.(R768W)	[30]
***LCA5***	c.1151del	p.(P384Qfs*18)	[63,65]
***RPGRIP1***	c.3565C>T	p.(R1189*)	[31]
***TULP1***	c.1138A>G	p.(T380A)	[17,40,63]
***TULP1***	c.1466A>G	p.(K489R)	[39,40]

^#^In original description [64] this variant erroneously was indicated as c.3101T>C.

^$^In lymphoblast cells, 50% of the resulting mRNA contains a cryptic exon resulting in a predicted stop mutation and 50% of the mRNA is normal [28].

### *In silico* analysis

The pathogenicity index for the identified missense mutations was calculated *in silico* using Sorting Intolerant From Tolerant (SIFT) (http://sift.bii.a-star.edu.sg/), Mutation Taster (http://www.mutationtaster.org/), and Polymorphism Phenotyping V2 (PolyPhen-2) (http://genetics.bwh.harvard.edu/pph2/). The PhyloP score and Grantham distances were also recorded to check the nucleotide conservation and change in amino acid physiochemical properties. The frequency of the variant in the general population was determined using Exome Variant Server (EVS) (http://evs.gs.washington.edu/EVS/), 1000 genomes and our in-house mutation database, which contained exome sequence variant data of 2,096 persons with various human conditions. To assess the effect of a missense change on the protein structure of CNGA1 we used the HOPE server http://www.cmbi.ru.nl/hope/home).

## Results, Discussion and Conclusions

### Clinical analyses

Typical features of RP and LCA as described in S1 Table were observed in the corresponding families and probands. The fundus pictures of the probands from selected families are given in S1 Fig.

### Families F01 and F02; *AIPL1*

**Fig 2:**
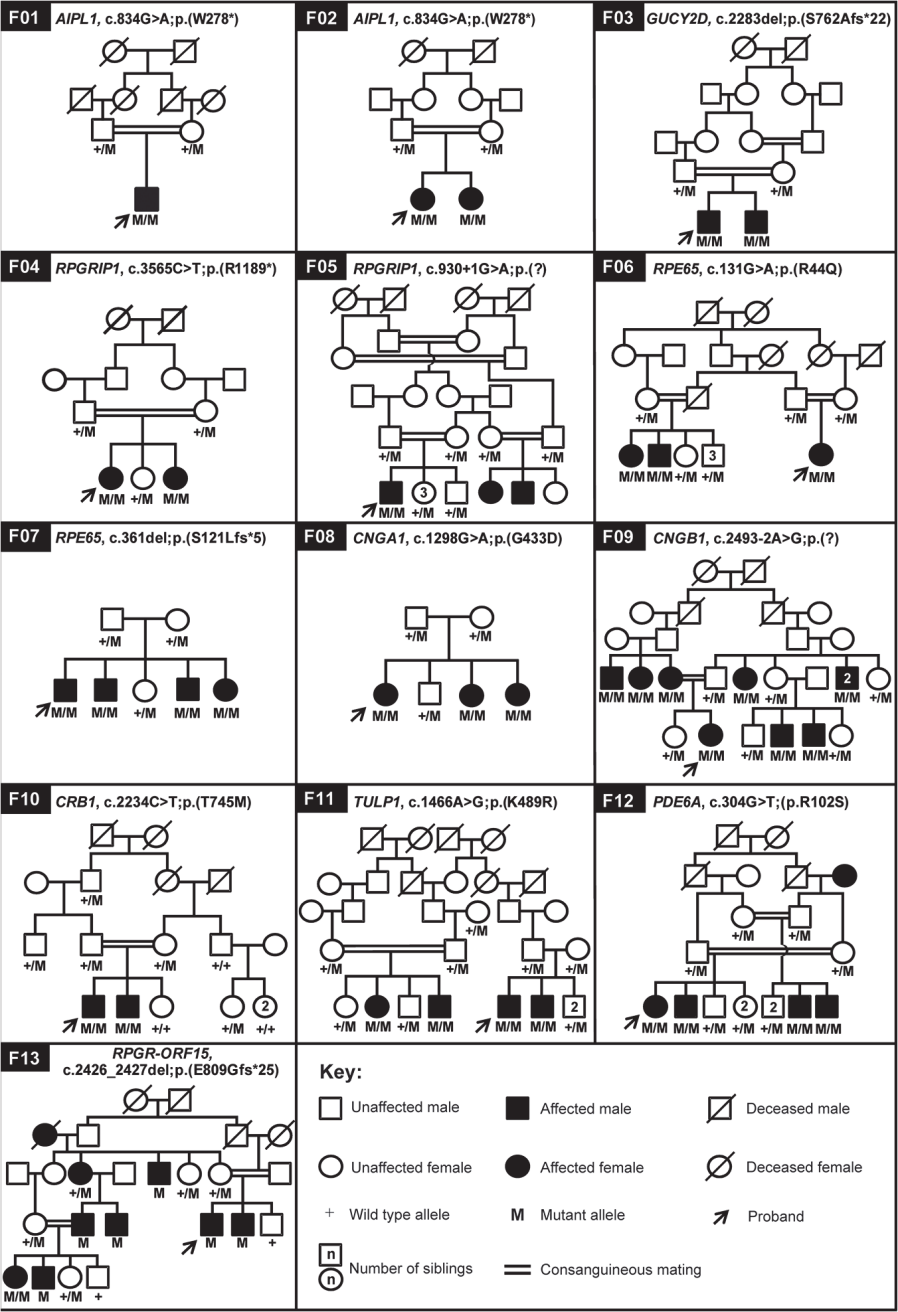
Pedigree structure and segregation analysis of disease causing variants in the IRD cohort. Arrows point to the probands.

The *AIPL1* exon 6 variation, c.834G>A; p.(W278*) [32] is a frequent LCA-associated variant worldwide and is responsible for 10% of the IRD cases reported so far in the Pakistani population [16]. Sanger sequencing of *AIPL1* exon 6 revealed two families with this mutation, which segregated with the disease in these families (Fig. 2, Table 1).

### Family F03; *GUCY2D*

Mutations in *GUCY2D* are known to cause LCA and CRD [33,34]. In our cohort of LCA, during targeted sequencing of exon 12 to search for c.2302C>T; p.(R768W) variant, we coincidentally identified a novel frame-shift mutation c.2283del; p.(S762Afs*22) in one LCA family (F03). Both affected siblings were homozygous for this 1-bp deletion whereas parents were heterozygous carriers (Fig. 2, Table 1).

### Families F04 and F05; *RPGRIP1*

Genetic linkage analysis revealed homozygous regions harboring the LCA-associated gene *RPGRIP1* in two of the families (F04 and F05) from the LCA panel. Upon sequencing *RPGRIP1* in family F04, a previously identified nonsense mutation c.3565C>T; p.(R1189*) [31] was identified in exon 22, which segregated with the phenotype in the family (Fig. 2, S1 Fig.). In family F05, a novel canonical splice donor site variation (c.930+1G>A; p.(?)) in intron 7 of the gene was identified. As the canonical splice donor site is affected, intron 7 retention or skipping of exon 7 in the mRNA is most plausible. Intron 7 retention would result in a frameshift that creates an early stop codon after 15 bp resulting in a truncated protein of 315 amino acid residues instead of the full length 1,286 amino acids (Fig. 2, Table 1). Skipping of exon 7 would not result in a frameshift but a deletion of 8 amino acid residues that might affect the three dimensional structure and thereby the function of the protein.

### Family F06 and F07; *RPE65*

The SNP array data of families F06 and F07 were analyzed to identify homozygous regions carrying the genes of interest. In both families, *RPE65* was identified in one of the largest homozygous regions. Upon sequencing, the most recurrent mutations, c.131G>A; p.(R44Q) [35] and 361del; p.(S121Lfs*6) [36], were identified in a homozygous state in all affected persons of families F06 and F07, respectively (Fig. 2, S1 Fig., Table 1, S2 Table).

### Family F08; *CNGA1*

Homozygosity mapping data of family F08 revealed the arRP-associated gene *CNGA1* in the largest homozygous region of ~19 Mb. Upon Sanger sequencing a novel missense mutation c.1298G>A; p.(G433D) was identified in the proband. This variant is not only absent in EVS and 1000 genomes public mutation databases, but also in our in-house WES database as well as from 90 ethnicity-matched healthy controls. Segregation analysis indicated that the mutation is present homozygously in affected individuals of the family whereas the normal individuals are heterozygous carriers (Fig. 2, Table 1). *In silico* analysis supported the pathogenicity of the mutation (S2 Table). The highly conserved non-polar glycine residue at position 433 is substituted by the charged aspartate, a bigger sized amino acid that is also less flexible than glycine. The wild type residue is predicted to be buried in a coiled region on the cytoplasmic face of the ion transport domain. The 433D residue can create structural instability and can affect the ion transport function of the protein [37].

### Family F09; *CNGB1*

The largest homozygous region of 6.5 Mb identified in family F09 harbored the arRP-associated gene *CNGB1*. Sequence analysis identified a novel homozygous canonical splice acceptor site mutation in intron 25 of *CNGB1*, c.2493-2A>G; p.(?), which segregated with the disease in the family (Fig. 2). This variant may result in exclusion of exon 26 from the transcript. The open reading frame would be shifted in the resulting transcript, leading to a truncated protein consisting of 831 amino acids (full length protein is 1,251 amino acids). In addition, due to this variation, a strong splice donor site is predicted that could result in the inclusion of a large part of intron 25 and exclusion of exon 26, which eventually would also lead to a premature stop codon (Fig. 2, Table 1, S1 Fig.).

### Family F10; *CRB1*

Homozygosity mapping positioned the arRP- and LCA-associated gene *CRB1* in one of the homozygous regions, which was shared between the affected individuals. A previously reported missense mutation, c.2234C>T; p.(T745M) [38], affecting the Laminin-G domain, was identified that segregated with the disease phenotype in the family (Fig. 2, Table 1, S2 Table).

### Family F11; *TULP1*

The largest homozygous region obtained for family F11 harbored *TULP1*, known to be associated with arRP and LCA. We identified a previously reported missense change, c.1466A>G; p.(K489R) [39,40], segregating with the disease phenotype in the family (Fig. 2, Table 1, S1 Fig., S2 Table).

### Family F12; *PDE6A*

Homozygosity mapping of family F12 revealed that *PDE6A* was in the largest region which spanned 6.5 Mb. The gene was sequenced and a previously identified missense mutation, c.304C>A; p.(R102S), was found to segregate with the disease phenotype in the family [41] (Fig. 2, Table 1, S2 Table).

### Family F13; *RPGR*

Family F13 was initially sampled as an autosomal recessive RP family but based on the pedigree structure (affected persons in multiple generations) and the fact that the far majority of the affected individuals are males, suggested X-linked inheritance. The analysis of SNP array data indeed pointed to *RPGR* as the candidate disease gene, as the region on the X-chromosome harboring this gene was found to be shared by all affected males. Sequence analysis of *RPGR* identified a 2-bp deletion, c.2426_2427del; p.(E809Gfs*25), in this family [42]. Interestingly, one of the affected females was also homozygous for the deletion, which is extremely rare in X-linked disorders (Fig. 2, Table 1) [43].

Inherited retinal diseases represent a diverse group of eye disorders that are heterogeneous both at the genotype and phenotype level. So far, mutations in 221 genes have been associated with syndromic and non-syndromic inherited retinal dystrophies, and still more are to be identified. This study underscores the genetic diversity of IRD as we report mutations in 10 different genes causing IRD in 13 families. To come to these results, we performed homozygosity mapping and candidate gene sequencing. This approach is successful for most of the consanguineous families. In outbred families this approach is only successful in a small proportion of families [13]. For such families, a pre-screening of frequently reported mutations can be an alternative method before starting with any high throughput analysis like next generation sequencing (NGS). To test this, we performed TSS of frequently found causative variants from seven IRD genes (Table 2) in 28 LCA probands. Despite being frequent in Pakistani and the Caucasian populations, most of them were not found in 25 families except the *AIPL1* variant p.(W278*), which was present in two families (F01 and F02). We were unable to find the recurrent exon 12 variant, p.(R768W) in *GUCY2D*. However, in the same exon, a novel variant (p.(S762Afs*22)) was identified in the proband of family F03. As we were able to solve only three families using TSS, this does not seem to be the best approach. For other populations this approach only makes sense if the frequent population-specific mutations are known.

Basic research in genetics has not only elucidated the underlying mutations in the causative genes but also provided initial information helpful for designing gene therapy. It has been estimated that 81.5% of all the gene therapy trials in the world are focused on cancer, cardiovascular diseases and monogenic inherited disorders. Other broadly targeted areas for gene therapy include infectious diseases, neurological disorders, ocular diseases, inflammatory diseases and diseases such as chronic renal disease, diabetes, etc [44]. The pre-clinical studies in model organisms, before initiation of any human trials, have provided detailed information not only on the therapeutic efficacy but also about safety and toxicity issues. Moreover, choosing the right model organism, which can provide as much information as possible for human trials is equally important [45]. In case of retinal disease gene therapy trials, a number of successful animal models have been described, for example, *AIPL1*, *GUCY2D*, *RPGRIP1* and *TULP1* knock out mouse models have already been reported in which gene therapy was explored [46–49]. In addition to mouse models, dog models for *RPGR* and *RPGRIP1* gene therapy are also known [50]. Similarly, *PDE6A* and *PDE6B* gene therapy proof-of-principle in mouse models were reported by Wert et al. [51]. The delivery method of a recombinant gene construct is important. For example, AAV-based gene therapy has been shown to be successful in a CRD dog model and in humans with *RPE65*-associated LCA [52–57], as well as in choroideremia subjects [58]. The major limitation of AAV-vector based gene therapy is that these vectors cannot carry inserts larger than 4.9 kb, and therefore other methods, viral and non-viral, are needed. Other types of treatments are based on antisense oligonucleotides for *CEP290*-associated retinal degeneration [59,60]. Besides these genetic approaches, an oral drug therapy based on 9-cis-retinoid was successful in persons with *RPE65* and *LRAT* mutations [61]. Thus, finding new associations for the IRD will not only add scientific knowledge but will also provide critical information for therapeutics.

In addition to gene therapy, another important aspect is genetic counseling. In the X-linked and autosomal recessive families, unaffected persons can be tested for carriership of the causal variants. Early genetic counseling may include advice on choosing appropriate studies and professions, improving their quality of life. Through proper genetic counseling the prevalence of the respective diseases in these families may decrease.

In conclusion, using homozygosity mapping, Sanger sequencing and TSS approaches we were able to identify the underlying genetic causes in 13 IRD families from Pakistan, and identified four novel variations in *CNGA1*, *CNGB1*, *GUCY2D* and *RPGRIP1* in four different families.

## Supporting Information

S1 Fig Fundus photographs of selected probands from families F04, F06, F09 and F11. Arrows mark the vessel attenuation, arrowheads represent changes in macula and a block arrows mark the pigmentary changes.
(TIF)

S1 Table Overview of the Pakistani IRD cohort and clinical characteristics. (DOCX)

S2 Table *In silico* analysis of the identified missense mutations. (DOCX)

## References

[1] Hamel C. Retinitis pigmentosa. Orphanet J Rare Dis. 2006;1: 40 17032466 10.1186/1750-1172-1-40 PMC1621055

[2] Nentwich MM, Rudolph G. Hereditary retinal eye diseases in childhood and youth affecting the central retina. Oman J Ophthalmol. 2013;6: S18–S25. 10.4103/0974-620X.122290 24391367 PMC3872838

[3] Rivolta C, Sharon D, DeAngelis MM, Dryja TP. Retinitis pigmentosa and allied diseases: numerous diseases, genes, and inheritance patterns. Hum Mol Genet. 2002;11: 1219–1227. 12015282 10.1093/hmg/11.10.1219

[4] Roosing S, van den Born LI, Hoyng CB, Thiadens AA, de Baere E, Collin RWJ, et al Maternal uniparental isodisomy of chromosome 6 reveals a TULP1 mutation as a novel cause of cone dysfunction. Ophthalmology. 2013;120: 1239–1246. 10.1016/j.ophtha.2012.12.005 23499059

[5] Estrada-Cuzcano A, Roepman R, Cremers FPM, den Hollander AI, Mans DA. Non-syndromic retinal ciliopathies: translating gene discovery into therapy. Hum Mol Genet. 2012;21: R111–124. 22843501 10.1093/hmg/dds298

[6] Robson AG, Michaelides M, Saihan Z, Bird AC, Webster AR, Moore AT, et al Functional characteristics of patients with retinal dystrophy that manifest abnormal parafoveal annuli of high density fundus autofluorescence; a review and update. Doc Ophthalmol. 2008;116: 79–89. 17985165 10.1007/s10633-007-9087-4 PMC2244701

[7] Ayuso C, Millan JM. Retinitis pigmentosa and allied conditions today: a paradigm of translational research. Genome Med. 2010;2: 34 10.1186/gm155 20519033 PMC2887078

[8] Jay M. On the heredity of retinitis pigmentosa. Br J Ophthalmol. 1982;66: 405–416. 7093178 10.1136/bjo.66.7.405 PMC1039814

[9] Adhi MI, Ahmed J. Frequency and clinical presentation of retinal dystrophies—A hospital based study. Pakistan J Ophthalmol. 2002;18: 106–110.

[10] Hamamy HA, Masri AT, Al-Hadidy AM, Ajlouni KM. Consanguinity and genetic disorders. Profile from Jordan. Saudi Med J. 2007;28: 1015–1017. 17603701

[11] Bittles A. Consanguinity and its relevance to clinical genetics. Clin Genet. 2001;60: 89–98. 11553039 10.1034/j.1399-0004.2001.600201.x

[12] Nirmalan PK, Krishnaiah S, Nutheti R, Shamanna BR, Rao GN, Thomas R. Consanguinity and eye diseases with a potential genetic etiology. Data from a prevalence study in Andhra Pradesh, India. Ophthalmic Epidemiol. 2006;13: 7–13. 16510341 10.1080/09286580500473795

[13] Collin RWJ, van den Born LI, Klevering BJ, de Castro-Miro M, Littink KW, Arimadyo K, et al High-resolution homozygosity mapping is a powerful tool to detect novel mutations causative of autosomal recessive RP in the Dutch population. Invest Ophthalmol Vis Sci. 2011;52: 2227–2239. 10.1167/iovs.10-6185 21217109

[14] Khan MI, Ajmal M, Micheal S, Azam M, Hussain A, Shahzad A, et al Homozygosity mapping identifies genetic defects in four consanguineous families with retinal dystrophy from Pakistan. Clin Genet. 2013;84: 290–293. 10.1111/cge.12039 23134348

[15] Littink KW, den Hollander AI, Cremers FPM, Collin RWJ. The power of homozygosity mapping: discovery of new genetic defects in patients with retinal dystrophy. Adv Exp Med Biol. 2012;723: 345–351. 10.1007/978-1-4614-0631-0_45 22183352

[16] Khan MI, Azam M, Ajmal M, Collin RWJ, den Hollander AI, Cremers FPM, et al The molecular basis of retinal dystrophies in Pakistan. Genes. 2014;5: 176–195. 10.3390/genes5010176 24705292 PMC3978518

[17] Ajmal M, Khan MI, Micheal S, Ahmed W, Shah A, Venselaar H, et al Identification of recurrent and novel mutations in TULP1 in Pakistani families with early-onset retinitis pigmentosa. Mol Vis. 2012;18: 1226–1237. 22665969 PMC3365133

[18] Ajmal M, Khan MI, Neveling K, Khan YM, Ali SH, Ahmed W, et al Novel mutations in RDH5 cause fundus albipunctatus in two consanguineous Pakistani families. Mol Vis. 2012;18: 1558–1571. 22736946 PMC3380946

[19] Azam M, Collin RWJ, Khan MI, Shah ST, Qureshi N, Ajmal M, et al A novel mutation in GRK1 causes Oguchi disease in a consanguineous Pakistani family. Mol Vis. 2009;15: 1788–1793. 19753316 PMC2742643

[20] Azam M, Collin RWJ, Malik A, Khan MI, Shah ST, Shah AA, et al Identification of novel mutations in Pakistani families with autosomal recessive retinitis pigmentosa. Arch Ophthalmol. 2011;129: 1377–1378. 10.1001/archophthalmol.2011.290 21987686

[21] Azam M, Collin RWJ, Shah ST, Shah AA, Khan MI, Hussain A, et al Novel CNGA3 and CNGB3 mutations in two Pakistani families with achromatopsia. Mol Vis. 2010;16: 774–781. 20454696 PMC2862243

[22] Azam M, Khan MI, Gal A, Hussain A, Shah ST, Khan MS, et al A homozygous p.Glu150Lys mutation in the opsin gene of two Pakistani families with autosomal recessive retinitis pigmentosa. Mol Vis. 2009;15: 2526–2534. 19960070 PMC2787306

[23] Khan MI, Collin RWJ, Arimadyo K, Micheal S, Azam M, Qureshi N, et al Missense mutations at homologous positions in the fourth and fifth laminin A G-like domains of eyes shut homolog cause autosomal recessive retinitis pigmentosa. Mol Vis. 2010;16: 2753–2759. 21179430 PMC3003713

[24] Khan MI, Kersten FFJ, Azam M, Collin RWJ, Hussain A, Shah ST, et al CLRN1 mutations cause nonsyndromic retinitis pigmentosa. Ophthalmology. 2011;118: 1444–1448. 10.1016/j.ophtha.2010.10.047 21310491

[25] Bandah-Rozenfeld D, Collin RWJ, Banin E, van den Born LI, Coene KLM, Siemiatkowska AM, et al Mutations in IMPG2, encoding interphotoreceptor matrix proteoglycan 2, cause autosomal-recessive retinitis pigmentosa. Am J Hum Genet. 2010;87: 199–208. 10.1016/j.ajhg.2010.07.004 20673862 PMC2917719

[26] Mackay DS, Borman AD, Sui R, van den Born LI, Berson EL, Ocaka LA, et al Screening of a large cohort of Leber congenital amaurosis and retinitis pigmentosa patients identifies novel LCA5 mutations and new genotype-phenotype correlations. Hum Mutat. 2013;34: 1537–1546. 10.1002/humu.22398 23946133 PMC4337959

[27] Sambrook J, Russell DW. Molecular cloning: a laboratory manual Newyork: Cold Spring Harbor Laboratory Press; 2001 pp. 44.

[28] den Hollander AI, Koenekoop RK, Yzer S, Lopez I, Arends ML, Voesenek KE, et al Mutations in the CEP290 (NPHP6) gene are a frequent cause of Leber congenital amaurosis. Am J Hum Genet. 2006;79: 556–561. 16909394 10.1086/507318 PMC1559533

[29] Li L, Xiao X, Li S, Jia X, Wang P, Guo X, et al Detection of variants in 15 genes in 87 unrelated Chinese patients with Leber congenital amaurosis. PLoS One. 2011;6: e19458 10.1371/journal.pone.0019458 21602930 PMC3094346

[30] Simonelli F, Ziviello C, Testa F, Rossi S, Fazzi E, Bianchi PE, et al Clinical and molecular genetics of Leber's congenital amaurosis: a multicenter study of Italian patients. Invest Ophthalmol Vis Sci. 2007;48: 4284–4290. 17724218 10.1167/iovs.07-0068

[31] Abu-Safieh L, Alrashed M, Anazi S, Alkuraya H, Khan AO, Al-Owain M, et al Autozygome-guided exome sequencing in retinal dystrophy patients reveals pathogenetic mutations and novel candidate disease genes. Genome Res. 2013;23: 236–247. 10.1101/gr.144105.112 23105016 PMC3561865

[32] Sohocki MM, Bowne SJ, Sullivan LS, Blackshaw S, Cepko CL, Payne AM, et al Mutations in a new photoreceptor-pineal gene on 17p cause Leber congenital amaurosis. Nat Genet. 2000;24: 79–83. 10615133 10.1038/71732 PMC2581448

[33] Kelsell RE, Evans K, Gregory CY, Moore AT, Bird AC, Hunt DM. Localisation of a gene for dominant cone-rod dystrophy (CORD6) to chromosome 17p. Hum Mol Genet. 1997;6: 597–600. 9097965 10.1093/hmg/6.4.597

[34] Perrault I, Rozet JM, Calvas P, Gerber S, Camuzat A, Dollfus H, et al Retinal-specific guanylate cyclase gene mutations in Leber's congenital amaurosis. Nat Genet. 1996;14: 461–464. 8944027 10.1038/ng1296-461

[35] Simovich MJ, Miller B, Ezzeldin H, Kirkland BT, McLeod G, Fulmer C, et al Four novel mutations in the RPE65 gene in patients with Leber congenital amaurosis. Hum Mutat. 2001;18: 164–168. 11462243 10.1002/humu.1168

[36] Coppieters F, De Baere E, Leroy B. Development of a next-generation sequencing platform for retinal dystrophies, with LCA and RP as proof of concept. Bull Soc Belge Ophtalmol. 2011: 59–60. 21560862

[37] Venselaar H, Te Beek TA, Kuipers RK, Hekkelman ML, Vriend G. Protein structure analysis of mutations causing inheritable diseases. An e-Science approach with life scientist friendly interfaces. BMC Bioinformatics. 2010;11: 548 10.1186/1471-2105-11-548 21059217 PMC2992548

[38] den Hollander AI, ten Brink JB, de Kok YJM, van Soest S, van den Born LI, van Driel MA, et al Mutations in a human homologue of Drosophila crumbs cause retinitis pigmentosa (RP12). Nat Genet. 1999;23: 217–221. 10508521 10.1038/13848

[39] Gu S, Lennon A, Li Y, Lorenz B, Fossarello M, North M, et al Tubby-like protein-1 mutations in autosomal recessive retinitis pigmentosa. Lancet. 1998;351: 1103–1104. 9660588 10.1016/S0140-6736(05)79384-3

[40] Iqbal M, Naeem MA, Riazuddin SA, Ali S, Farooq T, Qazi ZA, et al Association of pathogenic mutations in TULP1 with retinitis pigmentosa in consanguineous Pakistani families. Arch Ophthalmol. 2011;129: 1351–1357. 10.1001/archophthalmol.2011.267 21987678 PMC3463811

[41] Dryja TP, Rucinski DE, Chen SH, Berson EL. Frequency of mutations in the gene encoding the alpha subunit of rod cGMP-phosphodiesterase in autosomal recessive retinitis pigmentosa. Invest Ophthalmol Vis Sci. 1999;40: 1859–1865. 10393062

[42] Vervoort R, Lennon A, Bird AC, Tulloch B, Axton R, Miano MG, et al Mutational hot spot within a new RPGR exon in X-linked retinitis pigmentosa. Nat Genet. 2000;25: 462–466. 10932196 10.1038/78182

[43] Mendoza-Londono R, Hiriyanna KT, Bingham EL, Rodriguez F, Shastry BS, Rodriguez A, et al A Colombian family with X-linked juvenile retinoschisis with three affected females finding of a frameshift mutation. Ophthalmic Genet. 1999;20: 37–43. 10415464 10.1076/opge.20.1.37.2299

[44] Ginn SL, Alexander IE, Edelstein ML, Abedi MR, Wixon J. Gene therapy clinical trials worldwide to 2012—an update. J Gene Med. 2013;15: 65–77. 10.1002/jgm.2698 23355455

[45] Roosing S, Thiadens AAHJ, Hoyng CB, Klaver CCW, den Hollander AI, Cremers FPM. Causes and consequences of inherited cone disorders. Prog Retin Eye Res. 2014;42: 1–26. 10.1016/j.preteyeres.2014.05.001 24857951

[46] Lheriteau E, Petit L, Weber M, Le Meur G, Deschamps JY, Libeau L, et al Successful gene therapy in the RPGRIP1-deficient dog: a large model of cone-rod dystrophy. Mol Ther. 2014;22: 265–277. 10.1038/mt.2013.232 24091916 PMC3918913

[47] Beltran WA, Cideciyan AV, Lewin AS, Iwabe S, Khanna H, Sumaroka A, et al Gene therapy rescues photoreceptor blindness in dogs and paves the way for treating human X-linked retinitis pigmentosa. Proc Natl Acad Sci U S A. 2012;109: 2132–2137. 10.1073/pnas.1118847109 22308428 PMC3277562

[48] Boye SL, Peshenko IV, Huang WC, Min SH, McDoom I, Kay CN, et al AAV-mediated gene therapy in the guanylate cyclase (RetGC1/RetGC2) double knockout mouse model of Leber congenital amaurosis. Hum Gene Ther. 2013;24: 189–202. 10.1089/hum.2012.193 23210611 PMC3581260

[49] Ku CA, Chiodo VA, Boye SL, Goldberg AF, Li T, Hauswirth WW, et al Gene therapy using self-complementary Y733F capsid mutant AAV2/8 restores vision in a model of early onset Leber congenital amaurosis. Hum Mol Genet. 2011;20: 4569–4581. 10.1093/hmg/ddr391 21880665 PMC3209828

[50] Guyon R, Pearce-Kelling SE, Zeiss CJ, Acland GM, Aguirre GD. Analysis of six candidate genes as potential modifiers of disease expression in canine XLPRA1, a model for human X-linked retinitis pigmentosa 3. Mol Vis. 2007;13: 1094–1105. 17653054 PMC2779147

[51] Wert KJ, Davis RJ, Sancho-Pelluz J, Nishina PM, Tsang SH. Gene therapy provides long-term visual function in a pre-clinical model of retinitis pigmentosa. Hum Mol Genet. 2013;22: 558–567. 10.1093/hmg/dds466 23108158 PMC3542865

[52] Maguire AM, Simonelli F, Pierce EA, Pugh EN Jr, Mingozzi F, Bennicelli J, et al Safety and efficacy of gene transfer for Leber's congenital amaurosis. N Engl J Med. 2008;358: 2240–2248. 10.1056/NEJMoa0802315 18441370 PMC2829748

[53] Acland GM, Aguirre GD, Ray J, Zhang Q, Aleman TS, Cideciyan AV, et al Gene therapy restores vision in a canine model of childhood blindness. Nat Genet. 2001;28: 92–95. 11326284 10.1038/ng0501-92

[54] Annear MJ, Mowat FM, Bartoe JT, Querubin J, Azam SA, Basche M, et al Successful gene therapy in older Rpe65-deficient dogs following subretinal injection of an adeno-associated vector expressing RPE65. Hum Gene Ther. 2013;24: 883–893. 10.1089/hum.2013.146 24028205

[55] Acland GM, Aguirre GD, Bennett J, Aleman TS, Cideciyan AV, Bennicelli J, et al Long-term restoration of rod and cone vision by single dose rAAV-mediated gene transfer to the retina in a canine model of childhood blindness. Mol Ther. 2005;12: 1072–1082. 16226919 10.1016/j.ymthe.2005.08.008 PMC3647373

[56] Bennicelli J, Wright JF, Komaromy A, Jacobs JB, Hauck B, Zelenaia O, et al Reversal of blindness in animal models of Leber congenital amaurosis using optimized AAV2-mediated gene transfer. Mol Ther. 2008;16: 458–465. 10.1038/sj.mt.6300389 18209734 PMC2842085

[57] Le Meur G, Stieger K, Smith AJ, Weber M, Deschamps JY, Nivard D, et al Restoration of vision in RPE65-deficient Briard dogs using an AAV serotype 4 vector that specifically targets the retinal pigmented epithelium. Gene Ther. 2007;14: 292–303. 17024105 10.1038/sj.gt.3302861

[58] MacLaren RE, Groppe M, Barnard AR, Cottriall CL, Tolmachova T, Seymour L, et al Retinal gene therapy in patients with choroideremia: initial findings from a phase 1/2 clinical trial. Lancet. 2014;383: 1129–1137. 10.1016/S0140-6736(13)62117-0 24439297 PMC4171740

[59] Collin RWJ, den Hollander AI, van der Velde-Visser SD, Bennicelli J, Bennett J, Cremers FPM. Antisense oligonucleotide (AON)-based therapy for Leber congenital amaurosis Caused by a frequent mutation in CEP290. Mol Ther Nucleic Acids. 2012;1: e14 10.1038/mtna.2012.3 23343883 PMC3381589

[60] Gerard X, Perrault I, Hanein S, Silva E, Bigot K, Defoort-Delhemmes S, et al AON-mediated exon skipping restores ciliation in fibroblasts harboring the common Leber congenital amaurosis CEP290 mutation. Mol Ther Nucleic Acids. 2012;1: e29 10.1038/mtna.2012.21 23344081 PMC3390222

[61] Koenekoop RK, Sui R, Sallum J, van den Born LI, Ajlan R, Khan A, et al Oral 9cis retinoid for childhood blindness due to Leber congenital amaurosis caused by RPE65 or LRAT mutations: an open-label phase 1b trial. Lancet. 2014;6736: 60153–60157. 10.1016/S0140-6736(14)60153-7 25030840

[62] Damji KF, Sohocki MM, Khan R, Gupta SK, Rahim M, Loyer M, et al Leber's congenital amaurosis with anterior keratoconus in Pakistani families is caused by the Trp278X mutation in the AIPL1 gene on 17p. Can J Ophthalmol. 2001;36: 252–259. 11548141 10.1016/s0008-4182(01)80018-1

[63] McKibbin M, Ali M, Mohamed MD, Booth AP, Bishop F, Pal B, et al Genotype-phenotype correlation for Leber congenital amaurosis in Northern Pakistan. Arch Ophthalmol. 2010;128: 107–113. 10.1001/archophthalmol.2010.309 20065226

[64] Khaliq S, Abid A, Hameed A, Anwar K, Mohyuddin A, Azmat Z, et al Mutation screening of Pakistani families with congenital eye disorders. Exp Eye Res. 2003;76: 343–348. 12573663 10.1016/s0014-4835(02)00304-4

[65] den Hollander AI, Koenekoop RK, Mohamed MD, Arts HH, Boldt K, Towns KV, et al Mutations in LCA5, encoding the ciliary protein lebercilin, cause Leber congenital amaurosis. Nat Genet. 2007;39: 889–895. 17546029 10.1038/ng2066

