# Supplementary figures and images for: Homozygosity Mapping and Targeted Sanger Sequencing Reveal Genetic Defects Underlying Inherited Retinal Disease in Families from Pakistan
Source: PLoS One. 2015 Mar 16;10(3):e0119806. doi: 10.1371/journal.pone.0119806 (PMC4361598; doi:10.1371/journal.pone.0119806)

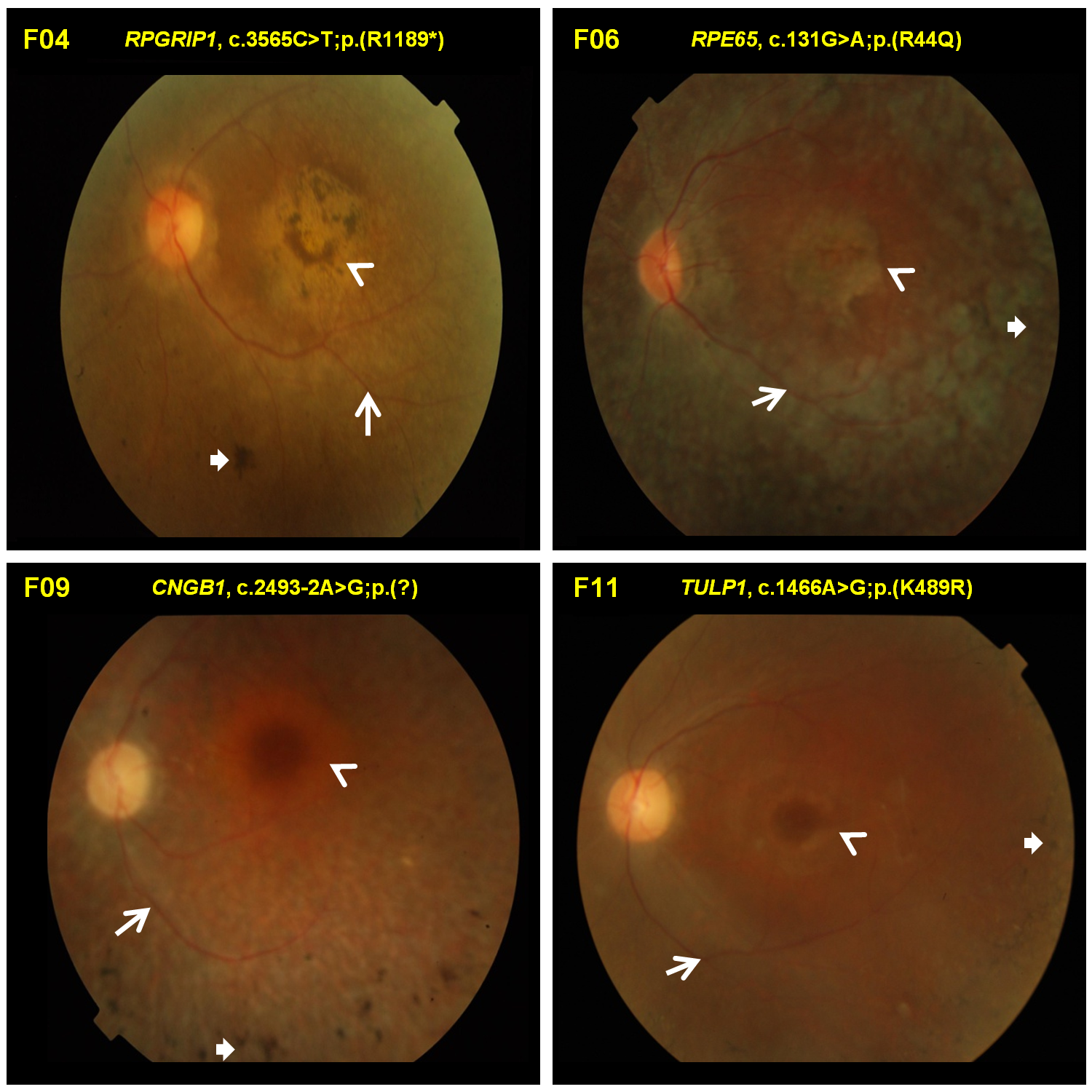

Supplement: S1 Fig — Arrows mark the vessel attenuation, arrowheads represent changes in macula and a block arrows mark the pigmentary changes. (TIF) [file pone.0119806.s001.tif]
