# Supplementary material for: Homozygosity Mapping and Targeted Sanger Sequencing Reveal Genetic Defects Underlying Inherited Retinal Disease in Families from Pakistan
Source: PLoS One. 2015 Mar 16;10(3):e0119806. doi: 10.1371/journal.pone.0119806 (PMC4361598; doi:10.1371/journal.pone.0119806)
Supplement: S1 Table — (DOCX) [file pone.0119806.s002.docx]

**S1 Table. Overview of the Pakistani IRD cohort and clinical characteristics.**

| **Phenotype** | **Clinical characteristics** | **Families analyzed** | **Solved families in this study** | **Total solved families** |
| --- | --- | --- | --- | --- |
| LCA* | Congenital blindness, nystagmus, sluggish/non-reactive pupil, myopic/hypermetropic fundus | 36 | 5 | 6 |
| RP | Peripheral vision loss, night blindness, bone spicules pigmentary deposits, reduced rod response | 38 | 8 | 26 |
| CD | Reduced central vision, poor color vision, photophobia, nystagmus, bull's eye macula | 2 | 0 | 2 |
| CSNB | Congenital stationary night blindness, whitish dots in fundus (FA), Mizuo phenomenon (Oguchi) | 5 | 0 | 4 |
| Total |  | 81 | 13 | 38^#^ |

*This includes 28 TSS analyzed families and 8 families analyzed using homozygosity mapping and Sanger sequencing of candidate genes.

^#^ 25 of 38 families are published elsewhere [1-12]. FA, fundus albipunctatus**.**

References:

1. Khan MI, Ajmal M, Micheal S, Azam M, Hussain A, Shahzad A, et al. Homozygosity mapping identifies genetic defects in four consanguineous families with retinal dystrophy from Pakistan. Clin Genet. 2013;84: 290-293.
2. Khan MI, Azam M, Ajmal M, Collin RWJ, den Hollander AI, Cremers FPM, et al. The molecular basis of retinal dystrophies in pakistan. Genes. 2014;5: 176-195.
3. Ajmal M, Khan MI, Micheal S, Ahmed W, Shah A, Venselaar H, et al. Identification of recurrent and novel mutations in TULP1 in Pakistani families with early-onset retinitis pigmentosa. Mol Vis. 2012;18: 1226-1237.
4. Ajmal M, Khan MI, Neveling K, Khan YM, Ali SH, Ahmed W, et al. Novel mutations in RDH5 cause fundus albipunctatus in two consanguineous Pakistani families. Mol Vis. 2012;18: 1558-1571.
5. Azam M, Collin RWJ, Khan MI, Shah ST, Qureshi N, Ajmal M, et al. A novel mutation in GRK1 causes Oguchi disease in a consanguineous Pakistani family. Mol Vis. 2009;15: 1788-1793.
6. Azam M, Collin RWJ, Malik A, Khan MI, Shah ST, Shah AA, et al. Identification of novel mutations in Pakistani families with autosomal recessive retinitis pigmentosa. Arch Ophthalmol. 2011;129: 1377-1378.
7. Azam M, Collin RWJ, Shah ST, Shah AA, Khan MI, Hussain A, et al. Novel CNGA3 and CNGB3 mutations in two Pakistani families with achromatopsia. Mol Vis. 2010;16: 774-781.
8. Azam M, Khan MI, Gal A, Hussain A, Shah ST, Khan MS, et al. A homozygous p.Glu150Lys mutation in the opsin gene of two Pakistani families with autosomal recessive retinitis pigmentosa. Mol Vis. 2009;15: 2526-2534.
9. Khan MI, Collin RWJ, Arimadyo K, Micheal S, Azam M, Qureshi N, et al. Missense mutations at homologous positions in the fourth and fifth laminin A G-like domains of eyes shut homolog cause autosomal recessive retinitis pigmentosa. Mol Vis. 2010;16: 2753-2759.
10. Khan MI, Kersten FFJ, Azam M, Collin RWJ, Hussain A, Shah ST, et al. CLRN1 mutations cause nonsyndromic retinitis pigmentosa. Ophthalmology. 2011;118: 1444-1448.
11. Bandah-Rozenfeld D, Collin RWJ, Banin E, van den Born LI, Coene KLM, Siemiatkowska AM, et al. Mutations in IMPG2, encoding interphotoreceptor matrix proteoglycan 2, cause autosomal-recessive retinitis pigmentosa. Am J Hum Genet. 2010;87: 199-208.
12. Mackay DS, Borman AD, Sui R, van den Born LI, Berson EL, Ocaka LA, et al. Screening of a large cohort of Leber congenital amaurosis and retinitis pigmentosa patients identifies novel LCA5 mutations and new genotype-phenotype correlations. Hum Mutat. 2013;34: 1537-1546.
