# Supplementary material for: Homozygosity Mapping and Targeted Sanger Sequencing Reveal Genetic Defects Underlying Inherited Retinal Disease in Families from Pakistan
Source: PLoS One. 2015 Mar 16;10(3):e0119806. doi: 10.1371/journal.pone.0119806 (PMC4361598; doi:10.1371/journal.pone.0119806)
Supplement: S2 Table — (DOCX) [file pone.0119806.s003.docx]

**S2 Table. *In silico* analysis of the identified missense mutations.**

| **Family** | **Gene** | **RefSeq Id** | **DNA variant** | **Protein variant** | **PhyloP** | **Grantham**  **distance** | **PolyPhen-2** | **SIFT** | **Allele frequency in EVS** | **References** |
| --- | --- | --- | --- | --- | --- | --- | --- | --- | --- | --- |
| F06 | *RPE65* | NM_000329.2 | c.131G>A | p.(R44Q) | 5.77 | 43 | Probably damaging (1.00) | Deleterious (0.02) | 0/13,006 | [1] |
| F08 | *CNGA1* | NM_00142564.1 | c.1298G>A | p.(G433D) | 5.77 | 94 | Probably damaging (1.00) | Deleterious (0.00) | 0/13,006 | This study |
| F10 | *CRB1* | NM_201253.2 | c.2234C>T | p.(T745M) | 4.16 | 81 | Probably damaging (1.00) | Deleterious (0.00) | 1/13,006 | [2] |
| F11 | *TULP1* | NM_003322.3 | c.1466A>G | p.(K489R) | 4.97 | 26 | Probably damaging (0.99) | Deleterious (0.00) | 0/13,006 | [3] |
| F12 | *PDE6A* | NM_000440.2 | c. 304C>A | p.(R102S) | 0.77 | 110 | Probably Damaging (1.00) | Deleterious (0.00) | 2/13,006 | [4] |

RefSeq, Reference sequence; DNA, Deoxyribonucleic acid; PhyloP, Phylogenetic p-value; PolyPhen 2, Polymorphism Phenotyping V 2; SIFT, Sorting Intolerant from Tolerant; EVS, Exome Variant Server.

References:

1. Coppieters F, De Baere E, Leroy B. Development of a next-generation sequencing platform for retinal dystrophies, with LCA and RP as proof of concept. Bull Soc Belge Ophtalmol. 2011: 59-60.
2. den Hollander AI, ten Brink JB, de Kok YJM, van Soest S, van den Born LI, van Driel MA, et al. Mutations in a human homologue of Drosophila crumbs cause retinitis pigmentosa (RP12). Nat Genet. 1999;23: 217-221.
3. Iqbal M, Naeem MA, Riazuddin SA, Ali S, Farooq T, Qazi ZA, et al. Association of pathogenic mutations in TULP1 with retinitis pigmentosa in consanguineous Pakistani families. Arch Ophthalmol. 2011;129: 1351-1357.
4. Dryja TP, Rucinski DE, Chen SH, Berson EL. Frequency of mutations in the gene encoding the alpha subunit of rod cGMP-phosphodiesterase in autosomal recessive retinitis pigmentosa. Invest Ophthalmol Vis Sci. 1999;40: 1859-1865.
